# Global and country-level data of the biodiversity footprints of 175 crops and pasture

**DOI:** 10.1016/j.dib.2021.106982

**Published:** 2021-03-20

**Authors:** Robert Beyer, Andrea Manica

**Affiliations:** a Department of Zoology, University of Cambridge^1^, Downing Street, Cambridge CB2 3EJ, United Kingdom; b Potsdam Institute for Climate Impact Research (PIK), Member of the Leibniz Association, Telegrafenberg A 31, 14473 Potsdam, Germany

**Keywords:** Biodiversity impact, Agriculture, Land use, Species richness, Threatened species, Range rarity

## Abstract

The destruction of natural habitat for cropland and pasture represents a major threat to global biodiversity. Despite widespread societal concern about biodiversity loss associated with food production, consumer access to quantitative estimates of the impact of crop production on the world's species has been very limited compared to assessments of other environmental variables such as greenhouse gas emissions or water use. Here, we present a consistent dataset of the biodiversity footprints of pasture and 175 crops at the global and national level. The data were generated by combining maps of the global distribution of agricultural areas in the year 2000 with spatially explicit estimates of the biodiversity loss associated with the conversion of natural habitat to farmland. Estimates were derived for three common alternative measures of biodiversity – species richness, threatened species richness, and range rarity – of the world's mammals, birds, and amphibians. Our dataset provides important quantitative information for food consumers and policy makers, allowing them to take evidence-based decisions to reduce the biodiversity footprint of global food production.

## Specifications Table

**Table:** 

Subject	Nature and Landscape Conservation
Specific subject area	Biodiversity impacts of global food production
Type of data	Table
How data were acquired	The data were generated by overlaying species distribution maps of mammals, birds, and amphibians with global maps of agricultural areas and yields
Data format	Analysed
Parameters for data collection	All species and crops available in the primary data sources were included.
Description of data collection	The data were generated based on existing species distribution and agricultural datasets
Data source location	Primary data sources: Species-specific range distribution and habitat data of birds [2], and mammal and amphibians [5], available from: http://datazone.birdlife.org/species/requestdis and https://www.iucnredlist.org, respectively. Global croplands and pastures [10], and crop-specific harvested areas [8], both available from: http://www.earthstat.org
Data accessibility	The data are available on the figshare repository: https://figshare.com/s/0695ac6fa97892225687.
Related Article	Beyer, R.M., & Manica, A. (2020). Historical and projected future range sizes of the world's mammals, birds, and amphibians. *Nature Communications, 11*(1), 1-8.

## Value of the Data

• Despite widespread consumer concern about the biodiversity footprint of global food production, consistent estimates of the impacts of different crops on the world's species are not available. Our dataset fills this gap by providing global and country-level impact estimates based on three common biodiversity measures.
• Our data provide important quantitative evidence to inform the decision-making of conservationists, policy makers, and food consumers, aiming to reduce food-related biodiversity impacts.
• Our estimates can be combined with crop-specific nutrient level data to rank crops and producing countries according to how efficiently they produce nutrients relative to the biodiversity footprint. This can facilitate the development of biodiversity-related food labelling systems, enabling consumers to reduce personal impacts and encourage shifts towards a sustainable food production.
• Our data also open the space for in-depth analyses of the heterogeneity in the local biodiversity footprint of specific crops within and across producing countries. A better understanding of these patterns will help inform where the future expansion of growing areas of specific crops should be prioritised in order to minimise biodiversity impacts.

## 1 Data Description

Our dataset (Table 1) contains the distributions of local biodiversity footprints across pastures and the harvested areas of 175 crops in the year 2000, based on three different biodiversity measures – species richness, threatened species richness, and range rarity –, at the global (cf. Fig. 4) and national level. Distributions are characterised in terms of 5th–95th distributional percentiles. In particular, for any given country and crop, the median value (50th percentile) of these distributions provides an estimate of the average number of species, average number of threatened species, and the average range rarity, that has been lost, compared to the scenario of natural habitat, on the growing areas of the crop of interest in the country of interest.

**Table 1:** Dataset specifications. Variable names ($b_{c,i}^{m}\left(p\right)$, ${\hat{b}}_{c,i}^{m}\left(p\right)$, $B_{c,i}^{m}$) are the ones used in the method description.

Dimension	Length	Values
Crops	176 (175 crops and pasture)	Abaca, Agave, …, Yautia, Pasture
Countries	166 (165 countries and world)	Afghanistan, Albania, …, Zimbabwe, World
Biodiversity measure	3	Species richness, Threatened species richness, Range rarity
Percentile	19	5th, 10th, …, 95th

Data variable	Dimensions	

Distribution of the local biodiversity footprint across agricultural areas, $b_{c,i}^{m}\left(p\right)$	176 × 166 × 3 × 19 (Crops and pasture × Countries × Biodiversity measures × Percentiles)	
Distribution of the local biodiversity footprint per unit of local crop yield across agricultural areas, ${\hat{b}}_{c,i}^{m}\left(p\right)$	175 × 166 × 3 × 19 (Crops × Countries × Biodiversity measures × Percentiles)	
Biodiversity footprint aggregated across agricultural areas, $B_{c,i}^{m}$	176 × 166 × 3 (Crops and pasture × Countries × Biodiversity measures)	

Analogous data are provided for the distributions of the ratio of local biodiversity footprints to local crop yields. These data are relevant, in particular, when linked to crop-specific nutritional data: multiplying them with by amount of a given nutrient in one unit of crop produce makes it possible to rank different crops according to how efficiently they provide the given nutrient relative to their biodiversity footprint.

In addition to the distributional data, our dataset contains spatially aggregated measures of the total biodiversity footprint of each crop in each country. These data make it possible, for example, to compare the contribution of specific crops or countries to the global biodiversity footprint of agriculture (cf. Fig. 5).

The three types of data are available as .xlsx files on the Figshare repository (https://figshare.com/s/0695ac6fa97892225687).

## 2 Experimental Design, Materials and Methods

### 2.1 Local biodiversity footprints of crops and pasture

We used the method previously described by [6] and [1] to estimate the geographical distribution of all known mammals, birds and amphibians under four different land cover types: natural vegetation, arable land, plantation, and pasture. In the following, we summarise the approach. We used species-specific extents of occurrence of mammals, birds, and amphibians [2,5], which we rasterised from their original spatial polygon format to a 5-arc-minute grid (~10 km at the equator). These data represent spatial envelopes of species’ maximum geographic ranges, and do not account for the distribution of natural or artificial land cover within these areas. Extents of occurrence were refined by incorporating species-specific habitat preferences [2,5], which include one or more biome categories in which each species is known to occur. In each grid cell contained within a given species’ extent of occurrence, the species was estimated as being present under natural vegetation if its list of habitat categories contained the local potential natural vegetation type, for which we used a 5-arc-minute global map [11]. In the same way, a species was estimated as being able to occur in a grid cell under arable land, plantation or pasture land cover, if its list of habitat categories included the relevant one of these three IUCN artificial land cover categories.

**Fig. 1:**
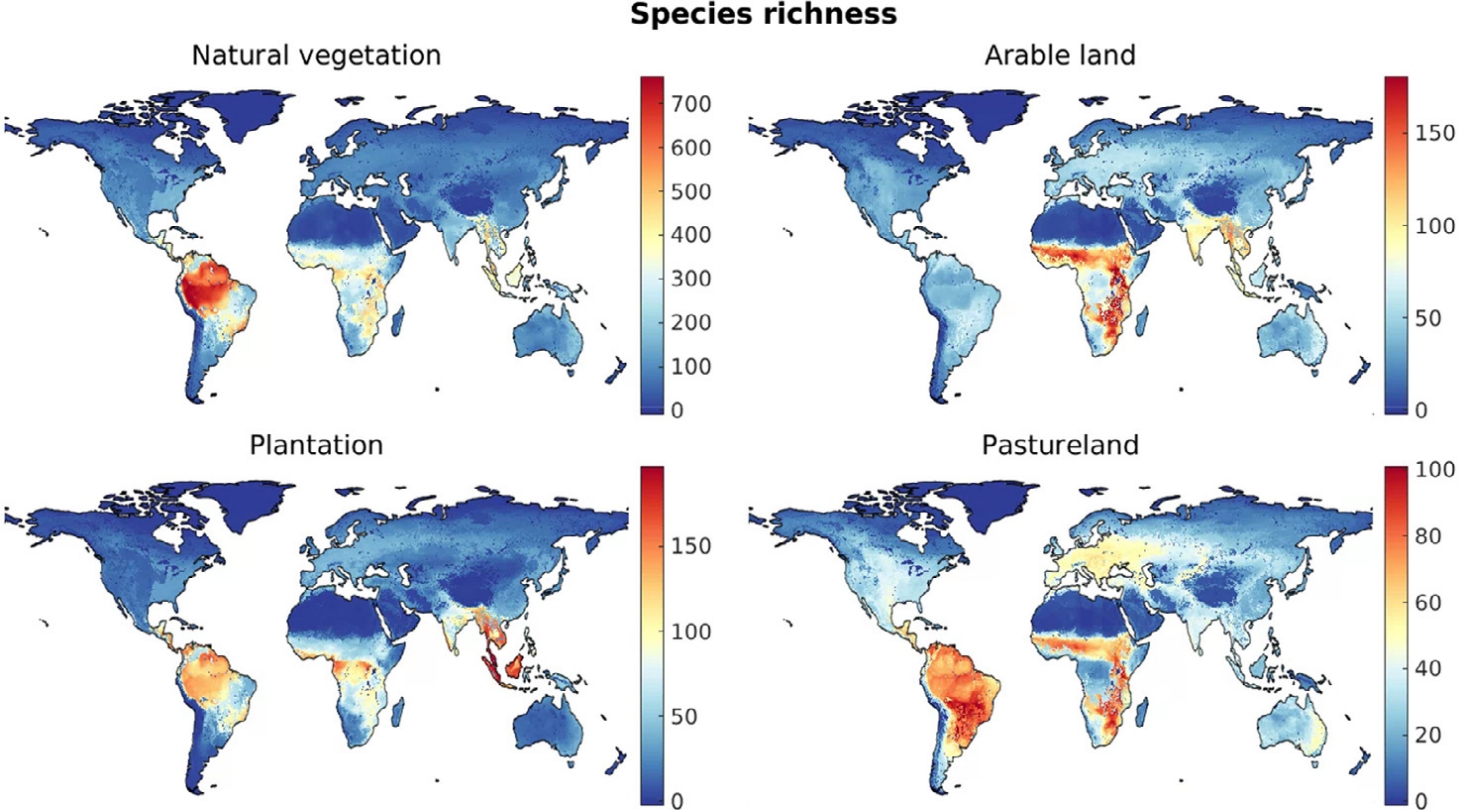
Global maps of species richness under different land cover types.

**Fig. 2:**
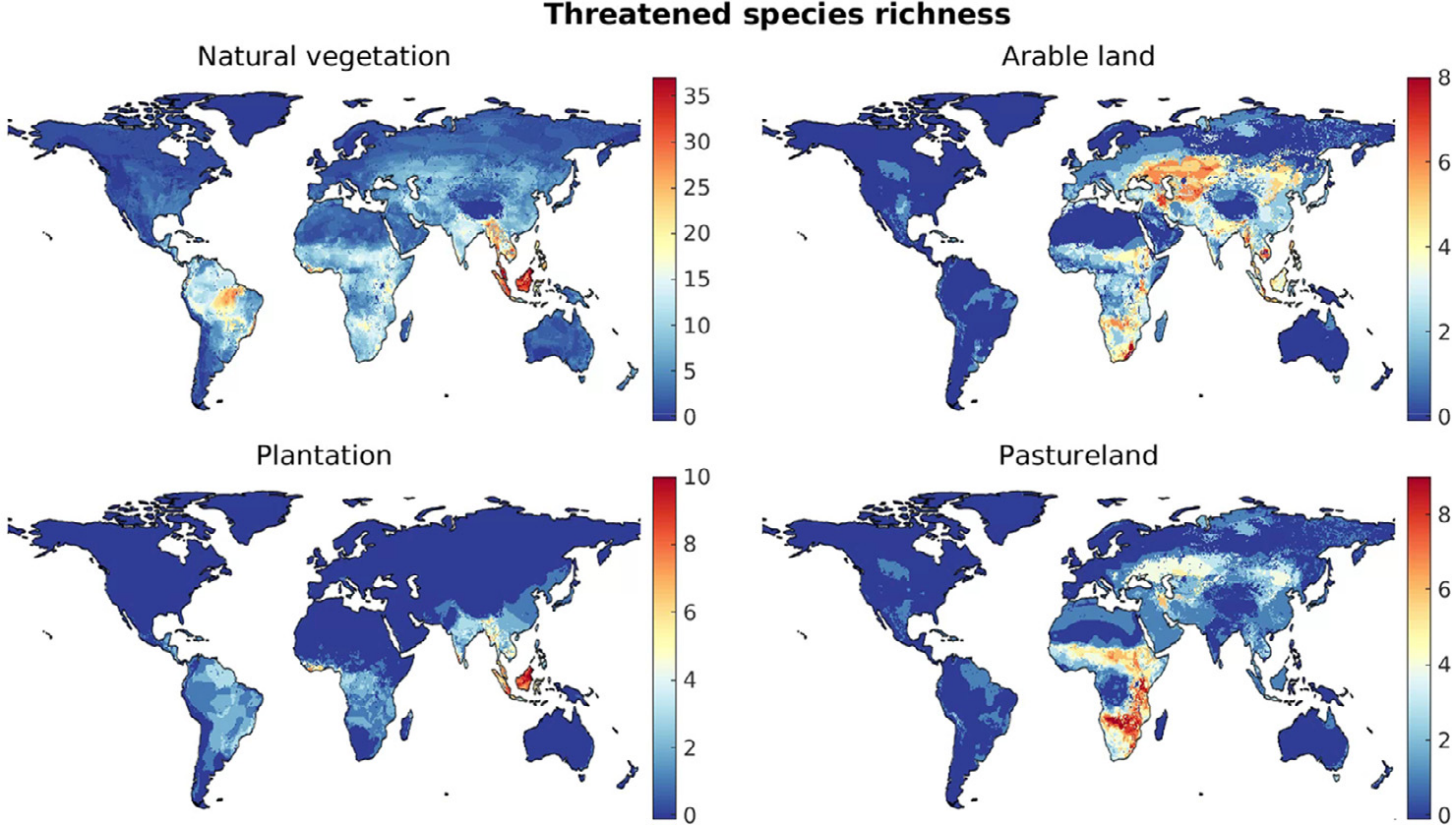
Global maps of threatend species richness under different land cover types.

**Fig. 3:**
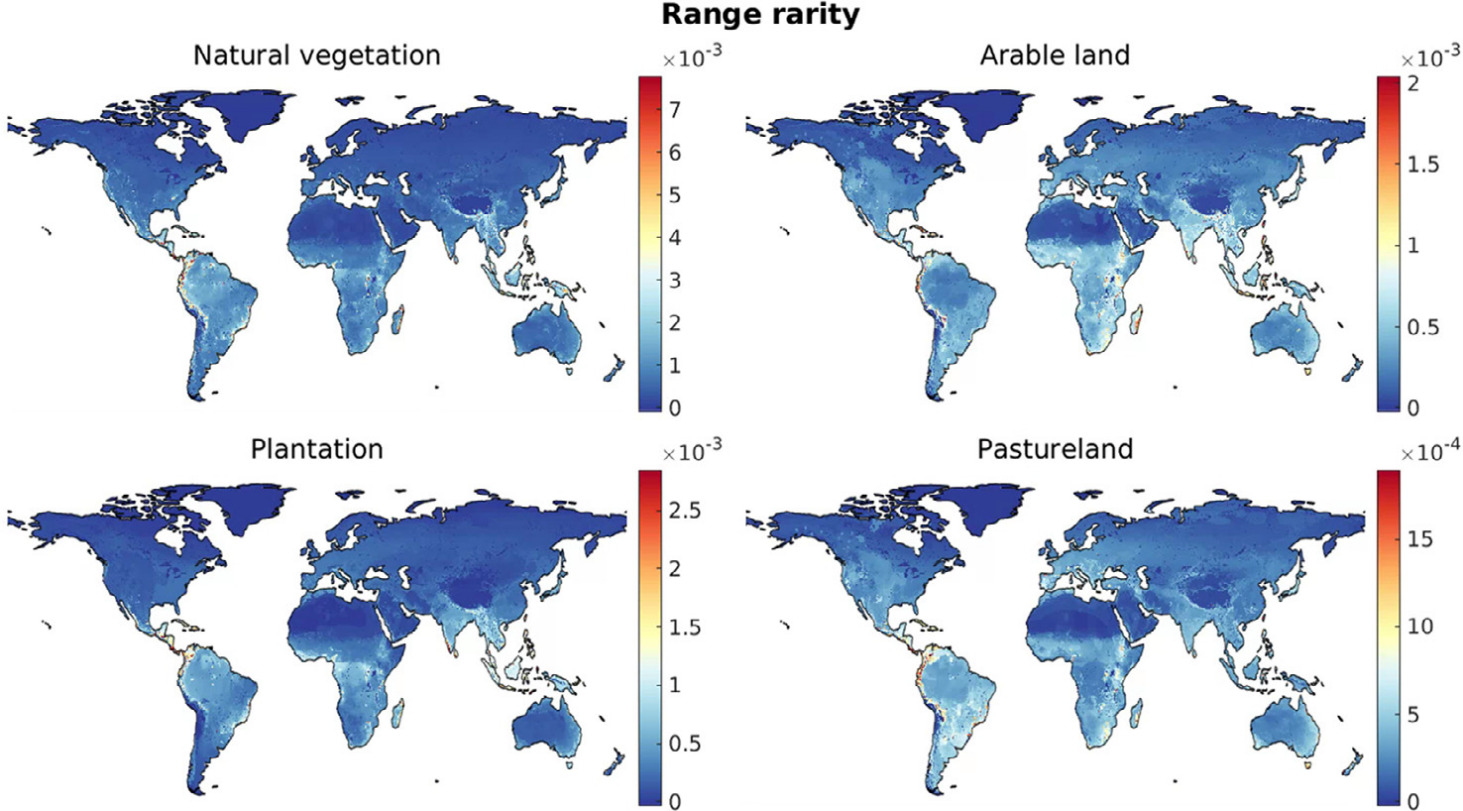
Global maps of range rarity (square-rooted) under different land cover types.

For the case of natural vegetation and for each of the three artificial land cover categories, maps of species richness, threatened species richness, and range rarity (Figs. 1–3) were derived as follows. Local species richness in a given grid cell is given by the number of species estimated as being present in the grid cell under the relevant land cover type. Threatened species richness was obtained in the same way but included only species whose Red List status is vulnerable, endangered, or critically endangered [2,5]. Range rarity [3] in a grid cell was calculated as the sum of the inverse natural range sizes of all species present in the cell under the relevant land cover. Thus, for this measure, species with a narrow geographic ranges are weighted more heavily than geographically widespread species [3].

Finally, for a given biodiversity measure m (representing species richness, threatened species richness, or range rarity) and a grid cell x, the local biodiversity footprint $\beta_{c}^{m}\left(x\right)$ associated with a crop c was defined as the difference between the local potential natural biodiversity and the biodiversity for the land cover type corresponding to the crop (either ‘arable land’ or ‘plantation’, as defined by [4]). Analogously, the local biodiversity footprint $\beta_{\text{Pasture}}^{m}\left(x\right)$ associated with pasture was defined as the difference between local potential natural biodiversity and biodiversity under pasture.

### 2.2 Crop- and pasture-specific distributions of biodiversity footprints at country level

**Fig. 4:**
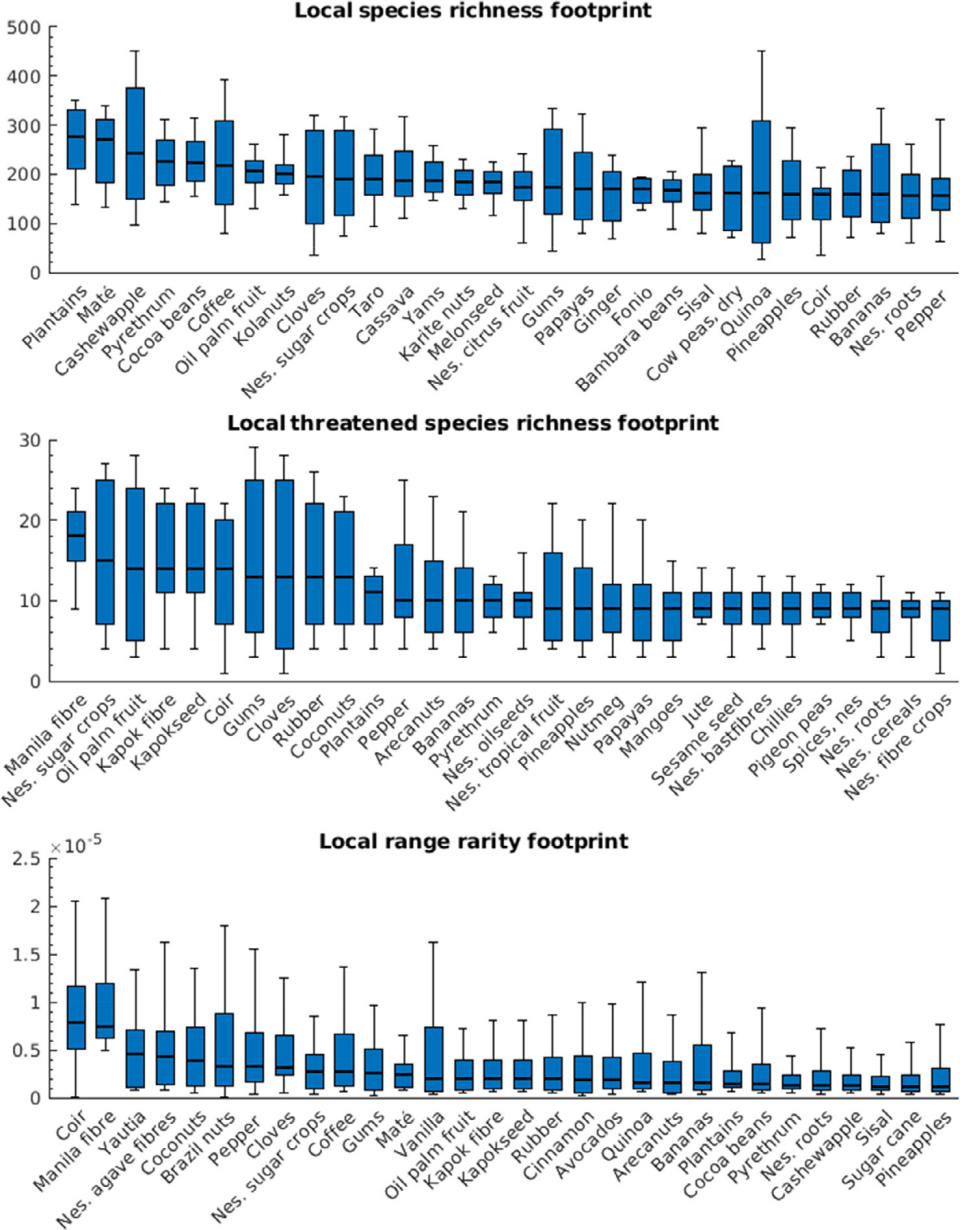
Distributions of local biodiversity footprints across global harvested areas of the 30 crops with the highest median value. (Nes. = not elsewhere specified, i.e., minor crop varieties.)

Based on the derived biodiversity footprint maps (Figs. 1–3), we first determined the distribution of biodiversity footprints across the harvested areas of each crop c. We denote by $H_{c}\left(x\right)$ the harvested area (in ha) in the year 2000 of a crop c in a 5-arc-minute grid cell x, which is available for 175 crops [8]. These maps of harvested areas represent the latest consistent global dataset containing all crops included in our dataset. More recent maps, based on different methods of spatial allocation of cropland, are available only for a much smaller number of crops; for consistency, we did not include these here, but used the 2000 data throughout our approach. We characterised the distribution of the biodiversity footprints across global harvested areas of crop c in terms of the 5th, 10th, ..., 95th percentile of the set of local biodiversity footprints, ${\left\{\beta_{c}^{m}\left(x\right)\right\}}_{x}$, where each element $\beta_{c}^{m}\left(x\right)$ was weighted by the appropriate local harvested area $H_{c}\left(x\right)$. We used the *wprctile* function in Matlab [7] to compute the percentiles for each crop, denoted $b_{c,\text{World}}^{m}\left(p\right)$ for a percentile p. For example, for m=speciesrichness, $b_{c,\text{World}}^{m}\left(50\right)$, i.e. the weighted median biodiversity footprints across the harvested areas of crop c, represents the number of species that are absent in a typical location where crop c is grown, compared to the scenario of potential natural vegetation. Fig. 4 visualises the derived $b_{c,\text{World}}^{m}\left(p\right)$ using boxplots for the 30 crops with the highest median biodiversity footprint across global harvested areas.

**Fig. 5:**
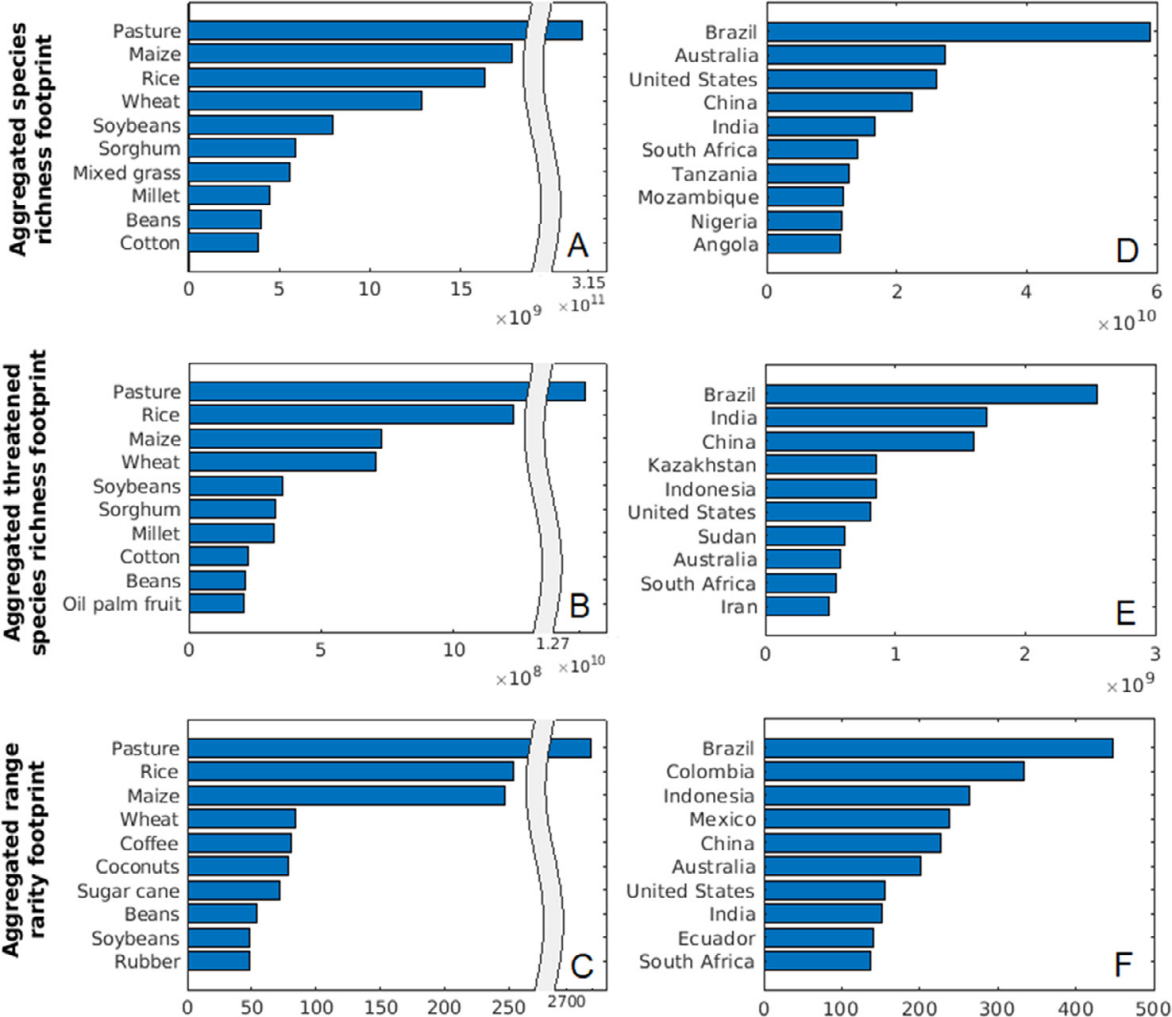
Spatially aggregated biodiversity footprints. A–C: Global footprints of pasture and the nine highest impact crops. D–F: Combined footprints of pasture and all crops in the ten highest impact countries.

Crop-specific percentiles of biodiversity footprints across the harvested areas of a cropc in a specific country i, denoted $b_{c,i}^{m}\left(p\right)$, were computed in the same way, but weights corresponding to grid cells located outside the country's borders [9] were set to zero.

Percentiles of biodiversity footprints across global pastures, $b_{\text{Pasture},\text{World}}^{m}\left(p\right),$ were computed analogously, based on the set of local biodiversity footprints of pasture ${\left\{\beta_{\text{Pasture}}^{m}\left(x\right)\right\}}_{x}$ and weights given by the set of local pasture areas (in ha), denoted ${\left\{A_{\text{pasture}}\left(x\right)\right\}}_{x}$, for the year 2000 [10]. Percentiles of biodiversity footprints across pastures in a given country i, denoted $b_{\text{Pasture},i}^{m}\left(p\right)$, were again computed by setting non-relevant weights to zero.

### 2.3 Crop-specific distributions of biodiversity footprints per unit yield at country level

In addition to estimating the distributions of local biodiversity footprints on agricultural areas, we used crop-specific global maps of fresh-weight yields (in Mg ha^−1^ year^−1^) for the year 2000 [8] to estimate the 5th, 10th, ..., 95th percentile of the crop- and country-specific biodiversity footprints per unit of crop yield across croplands, denoted ${\hat{b}}_{c,\text{World}}^{m}\left(p\right)$. For a given crop c, these were computed as the percentiles of the set ${\left\{\frac{\beta_{c}^{m}\left(x\right)}{Y_{c}\left(x\right).\;}\right\}}_{x}$ of local ratios of biodiversity footprint divided by local crop yields, $Y_{c}\left(x\right)$, of the crop c, weighted by the set of local harvested areas ${\left\{H_{c}\left(x\right)\right\}}_{x}$. Country-specific percentiles, ${\hat{b}}_{c,i}^{m}\left(p\right)$, were derived analogously. These data are relevant, in particular, when estimating how efficiently a cropc provides a certain nutrient relative to its biodiversity footprint. For example, multiplying ${\hat{b}}_{c,i}^{m}\left(50\right)$ by the amount of a certain nutrient in one unit of produce of crop c provides an estimate of the average biodiversity footprint associated with the production of one unit of the given nutrient from crop c grown in countryi.

### 2.4 Crop-specific total biodiversity footprints at country level

Thus far, we considered the distributions of (absolute and per-yield) local biodiversity footprints across agricultural areas. In addition, we derived spatially aggregated estimates of the biodiversity footprints of crops and pastures at country level by integrating local footprints over the relevant areas. For pasture, we used the physical area (in ha) covered by pasture in a grid cell x in the year 2000 [10], $A_{\text{Pasture}}\left(x\right)$, to define the spatially-aggregted global biodiversity footprint of pasture as

$$B_{\text{Pasture},\text{World}}^{m}=\sum_{x}A_{\text{Pasture}}\left(x\right)\cdot \beta_{\text{Pasture}}^{m}\left(x\right)$$

In the case of crops, we used the physical area (in ha) covered by cropland in a grid cell x in the year 2000 [10], $A_{\text{Cropland}}\left(x\right)$, and the harvested area (in ha) in the year 2000 of a crop c in a grid cell x [8], $H_{c}\left(x\right)$. Generally, $A_{\text{Cropland}}\left(x\right)$ is not the same as $\sum_{c}H_{c}\left(x\right),\;$ the sum of all local harvested areas. This is because $H_{c}\left(x\right)$ does not represent physical area but harvested area which increases if the crop is harvested multiple times per year [8]. We accounted for this by calculating the spatially-aggregted global biodiversity footprint of a crop c as

$$B_{c,\text{World}}^{m}=\sum_{\;x}A_{\text{Cropland}}\left(x\right)\cdot \frac{H_{c}\left(x\right)}{\sum_{\gamma }H_{\gamma }\left(x\right)}\cdot \beta_{c}^{m}\left(x\right).$$

Fig. 5A–C display aggregated footprints for pasture and the nine highest-impact crops.

Analogous data at country level, denoted $B_{\text{Pasture},i}^{m}$ and $B_{c,i}^{m}$ for a country i, were computed in the same way but the relevant sums did not include grid cells outside of country i. The total spatially aggregated biodiversity footprint of pasture and all crops, $B_{\text{Pasture},i}^{m}+\sum_{c}B_{c,i}^{m}$, is visualised in Fig. 5D–F for the ten countries for which this value is highest.

## CRediT Author Statement

**Robert Beyer:** Conceptualisation, Methodology, Formal analysis, Writing - Original Draft; **Andrea Manica:** Conceptualisation, Methodology, Writing - Review & Editing.

## Declaration of Competing Interest

The authors declare no conflict of interest.
